# Glutamic Acid Increased Methotrexate Polyglutamation and Cytotoxicity in a CCRF-SB Acute Lymphoblastic Leukemia Cell Line

**DOI:** 10.3390/medicina55120758

**Published:** 2019-11-26

**Authors:** Alma Mendoza-Santiago, Edgardo Becerra, Edith Garay, Moustapha Bah, Laura Berumen-Segura, Jesica Escobar-Cabrera, Abigail Hernández-Pérez, Guadalupe García-Alcocer

**Affiliations:** 1 Unidad de Investigación Genética, Facultad de Química, Universidad Autónoma de Querétaro, Centro Universitario, 76010 Querétaro, Mexico; almita_0223@hotmail.com (A.M.-S.); ebecerra1989@gmail.com (E.B.); lcbsq@yahoo.com (L.B.-S.); jeec82@yahoo.com.mx (J.E.-C.); 2 Laboratorio de Investigación en Productos Naturales, Facultad de Química, Universidad Autónoma de Querétaro, 76010 Querétaro, Mexico; edith@unam.mx (E.G.); abymez@hotmail.com (A.H.-P.); 3 Instituto de Neurobiología, Universidad Nacional Autónoma de México, 04510 Mexico City, Mexico; moubah@uaq.mx

**Keywords:** polyglutamates, cytotoxicity, polyglutamation, methotrexate

## Abstract

*Background and Objectives:* Acute lymphoblastic leukemia (ALL) is the most common type of cancer in childhood. The majority of patients respond to treatment, but those with resistant phenotypes suffer relapse or death. The antifolate methotrexate (MTX) is the most commonly used drug against ALL due to its efficacy. Once inside leukemic cells, MTX is metabolized into methotrexate polyglutamates (MTX-PG) by action of the enzyme folylpolyglutamate synthetase (FPGS), leading to a longer action compared to that of MTX alone. *Materials and Methods:* In this work, we demonstrated that the combination treatment of methotrexate and 5 and 10 mM glutamic acid could enhance methotrexate cytotoxicity in CCRF-SB (B-ALL) cells. In addition, MTX plus 20 mM glutamic acid was able to improve the synthesis of MTX-PG_5_. *Results:* All treatments induced an increase in FPGS expression compared to that of the control group. Furthermore, we detected different cellular expression patterns of FPGS in the different treatments. *Conclusion*: Based on these findings, we demonstrated that levels of methotrexate polyglutamates (MTX-PGs) could be a key determinant of methotrexate-induced cytotoxicity in CCRF-SB acute lymphoblastic leukemia cells.

## 1. Introduction

Acute lymphoblastic leukemia (ALL) is the most common type of cancer in childhood, with an incidence peak between 2 and 5 years of age [1]. Although the majority of patients with ALL respond well to therapy, a poor outcome remains for patients with resistant phenotypes as well as for those who suffer a relapse [2]. For this reason, novel strategies for the treatment of childhood ALL are needed to improve the effectiveness of the current chemotherapeutic drugs and thus increase the cure rate and reduce toxicity to non-malignant cells.

The main purpose of traditional chemotherapeutics is to interrupt the biochemical processes that are involved in cancer cell growth and proliferation. Methotrexate (MTX) is an agent that could be considered the backbone of ALL treatment because it is used throughout all therapy stages and intracranial hemorrhage has been reported after oral administration [2,3,4]. MTX is an antifolate that inhibits dihydrofolate reductase (DHFR), a key enzyme of the folic acid pathway that is required for the synthesis of RNA and DNA [5]. In addition, once inside mammalian cells, methotrexate acts as a substrate for the enzyme folylpolyglutamate synthetase (FPGS), which catalyses the addition of glutamate residues via γ-linkages to the end carboxyl group of methotrexate, leading to methotrexate polyglutamate (MTX-PG) synthesis [6].

The synthesis of MTX-PG has important consequences that enhance the cytotoxic action of MTX. First, MTX-PG is retained in cells for long intervals; this accumulation not only results in a sustained inhibition of the folate pathway but also increases the inhibitory effect against their target enzymes by more than 100-fold [7,8,9]. Furthermore, a comparison of MTX metabolism between normal and cancer cells revealed that MTX-PG synthesis was twofold greater in cancer cells, suggesting that MTX polyglutamation could be critical to its selective anti-leukemic effect [10].

The implications of polyglutamate formation are now being appreciated, so the levels of MTX-PG accumulation in lymphoblasts, both in vitro and in vivo, are measured by the HPLC technique to use its intracellular concentration as a predictor of treatment outcome in childhood acute lymphoblastic leukemia [11,12,13].

In previous reports, the synergism of MTX combined with other chemotherapeutic drugs, such as histone deacetylase inhibitors, and the extent to which these synergistic strategies promote the synthesis and accumulation of MTX-PG, as well as the cytotoxicity of MTX in ALL cell lines have been studied [8]. However, there have been no attempts to improve MTX polyglutamation through an agent that does not belong to the anticancer drug group.

Recently, it has been suggested that glutamic acid plays important roles in anticancer therapy, for example, when it is conjugated to chemotherapeutic agents such as all-trans retinoic acid (ATRA). ATRA is used in the treatment of acute promyelocytic leukemia and myelodysplastic syndrome. Two derivatives of ATRA (containing glutamic acid or its sodium salt), RAE and RAENa, have been synthesized, and they showed improved aqueous solubility and were more effective in mice bearing S-180 tumors and in childhood acute leukemia [14,15]. In the present study, we proposed glutamic acid as an adjuvant to increase MTX efficacy by promoting polyglutamate synthesis. The increase in MTX efficacy was evaluated and correlated with an increase in the cytotoxicity of lymphoblasts evaluated by the trypan blue exclusion test and with the level of MTX-PG formation measured by HPLC. Additionally, we performed immunocytochemistry studies to determine whether the treatments influenced the expression level of the glutamate transporter GLAST and the expression and distribution of the FPGS enzyme. Finally, we performed flow cytometry analysis to evaluate the effect of treatments on programmed cell death (apoptosis).

## 2. Materials and Methods

### 2.1. Maintenance of Cell Culture

CCRF-SB human acute lymphoblastic leukemia cells (ATCC® CCL120^TM^, Manassas, VA, USA) were grown as suspension cultures in RPMI-1640 medium (ATCC) supplemented with 10% foetal bovine serum (ATCC) and 1% penicillin-streptomycin (ATCC). Leukemia cells were routinely subcultured twice weekly and were maintained in an incubator with 5% CO_2_ and a humidified atmosphere at 37 °C.

### 2.2. Concentration–Response Curve for Methotrexate Citotoxicity

Cells in the exponential growth phase were counted by the trypan blue exclusion test and seeded in fresh media at a density of 2.5 × 10^5^ cells/mL in a 12-well plate. Twenty-four hours after cell plating, the cells were exposed to methotrexate (Laboratorios PiSA, México City, Mexico). MTX was dissolved in water at 1 mg/mL. The stock solution was diluted further in RPMI-1640 to the final concentration required. To ensure that a complete sigmoidal cytotoxicity concentration curve could be observed, the following drug concentrations were studied: 0.01, 0.03, 0.05, 0.1, 0.5, 1, 5, and 10 μM. The IC_50_ and IC_25_ are the concentrations of the drug required to reduce cell viability by 50 and 25%, respectively, and were calculated by fitting the experimental data to a three-parameter logistic equation (GraphPad Prism 6, San Diego, CA, USA). This model assumes that the dose–response curves have a standard slope equal to a hill slope (or slope factor) of −1.0.

Model:

$$Y=\mathrm{Bottom}+\;\frac{\left(\mathrm{Top}-\mathrm{Bottom}\right)}{1+10^{\left(x-{\mathrm{LogIC}}_{50}\right)}}$$

where IC_50_ or IC_25_ is the concentration that provokes a response halfway between the maximal (Top) response and the maximally inhibited (Bottom) response.

### 2.3. Drug Combination Treatment

Cells were treated with different drug combinations as indicated in Table 1.

### 2.4. HPLC Analysis of MTX Polyglutamate Formation

Cells previously treated with 20 mM glutamic acid and MTX or only with MTX were centrifuged at 125× *g* for 5 min, and the medium was removed. The cell pellet was washed twice in 5 mL of PBS followed by 2 mL of PBS, and the final cell pellet was lysed with 300 μL of ice-cold 0.2 M perchloric acid (Sigma-Aldrich, St. Louis, MO, USA). Samples were vortexed briefly and left on ice for 5 min before centrifugation at 6700× *g* for 2 min. The resulting supernatant was removed and pipetted directly onto 200 mg of potassium bicarbonate (Sigma) and left to stand on ice for 2 min. The neutralized cell extract was centrifuged again at 6700× *g* for 2 min to remove any remaining potassium bicarbonate and the potassium perchlorate that had formed, and the final supernatant was removed and stored at −20 °C until analysis by high-performance liquid chromatography. MTX polyglutamates were quantitated as described previously [11]. HPLC analysis was performed in a Waters chromatograph (Waters, Milford, MA, USA) equipped with a Waters 600 quaternary pump and a Rheodyne 7725 manual injector (Waters). MTX-PG_3-5_ were separated on a Zorbax Eclipse XDB-C18, 4.6 × 150 mm, 3.5 μm column (Agilent Technologies, Santa Clara, CA, USA). The mobile phase used with this chromatographic system consisted of Solvent A—10 mM sodium phosphate buffer pH 6.2 with 1.5 mL/L 30% H_2_O_2_—and Solvent B—20% acetonitrile with 1.5 mL/L 30% H_2_O_2_. The system was operated at a flow rate of 0.6 mL/min with a gradient elution programme of 10% B to 55% B in 15 min, followed by an isocratic hold for 5 min. The column was allowed to re-equilibrate for 10 min at the initial conditions. MTX-PGs were detected using a Waters model 2998 multiwavelength diode-array detector. MTX-PG quantification was achieved using the area under the curve of MTX-PG_3-5_ standards (Schirks Laboratories, Jona, Switzerland), and the results were expressed as pmol of MTX-PGs/10^7^ cells.

### 2.5. Immunocytochemical Analysis of FPGS Enzyme and GLAST Transporter

Cells previously treated with 10 or 20 mM glutamic acid and MTX or only with MTX were washed three times with PBS, and the peroxidase activity was quenched with a PBS/1% H_2_O_2_ solution for 1 h. The cells were then washed and blocked with 3% milk (BIO-RAD, Irvine, CA, USA) in PBT for 2 h. Following blocking, the cells were incubated with the rabbit polyclonal anti-FPGS antibody (Santa Cruz Biotechnology, Inc., Santa Cruz, CA, USA) or rabbit polyclonal anti-GLAST antibody (Santa Cruz Biotechnology) in 1% milk/PBT/ overnight at 4 °C. The cells were then washed and incubated with the secondary goat anti-rabbit IgG-B antibody (1:2000, Santa Cruz Biotechnology, Inc., Santa Cruz, CA, USA) in PBT. Following secondary antibody incubation, cells were washed with PBT and incubated in an avidin–biotin complex solution (Vector Laboratories Inc., Burlingame, CA, USA) in the dark for 30 min. Finally, the cells were washed with PBS, and the stain was developed with DAB (Bio-Rad, Irvine, CA, USA) in the dark until the signal was detected. Once DAB staining was visible, the reaction was blocked by adding PBS to the cells. The cells were evaluated with a light microscope under a 40× objective (Axioskop 2 plus ZEISS, Jena, Germany).

### 2.6. Assessment of Apoptosis with Annexin-V Staining

Apoptosis was assessed by Annexin V-fluorescein isothiocyanate and propidium iodine staining (apoptotic annexin V-IP^+^) (BD Pharmingen, San Diego, CA, USA) according to the manufacturer’s instructions. The percentage of surviving cells was determined by flow cytometry analysis using the BD FACSVerse system (BD Biosciences, San Jose, CA, USA).

### 2.7. Glutamate Uptake

MTX-dependent glutamate transport was measured as described by [16]. Briefly, cells previously treated with 5 or 10 mM glutamic acid and 0.13 μM MTX or only with 0.13 or 0.26 μM MTX were centrifuged at 125× *g* for 5 min, and the medium was removed. The cell pellet was washed once in 1 mL of an isosmotic solution. The washed cells were suspended in 0.5 mL of the isosmotic solution. The flux experiment was initiated by the addition of 1 μL of 1 mCi (37 MBq) L-[3,4-3H]-glutamic acid (PerkinElmer, Waltham, MA, USA). The incubation was stopped after 15 min by the addition of 1 mL of ice-cold PBS and washed three times with PBS. The cells were lysed with 300 μL of ice-cold 0.2 M perchloric acid (Sigma). Samples were vortexed briefly and left on ice for 5 min before centrifugation at 6700× *g* for 10 min. Radioactivity of the supernatants was determined with a liquid scintillation counter.

### 2.8. Statistical Analysis

Differences in mean values were evaluated using a one-way ANOVA with Tukey’s test to determine differences among treatment groups. *p* < 0.05 was regarded as statistically significant. All data are given as the mean ± standard deviation. Statistical analysis was performed by Graphpad prism 6.0 software, San Diego, CA, USA.

## 3. Results

### 3.1. IC_50_ of MTX 

**Figure 1:**
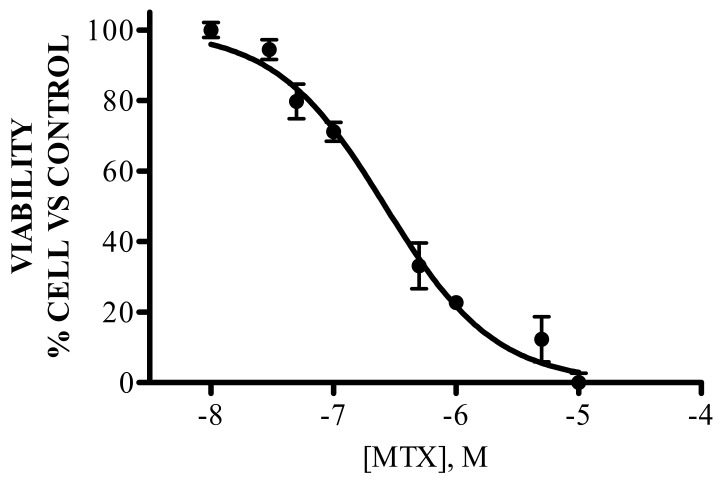
Concentration–response curve of methotrexate for the CCRF-SB cell line. This figure shows the percentage of cellular viability as a function of methotrexate concentration (M). Viability is expressed as a percentage of the viability of the untreated cells. Data are presented as the mean ± standard deviation, and they are representative of three independent sets of experiments.

To determine the half maximal inhibitory concentration of MTX, which was used in successive experiments, eight drug concentrations between 0.01 and 10 μM were evaluated. By fitting data through a three-parameter logistic equation, we obtained a representative dose–response curve, as shown in Figure 1. This graph was used to calculate the IC_50_ value for the CCRF-SB cell line, which in this case, was 0.2108–0.3333 μM.

### 3.2. Glutamic Acid Increases the Intracellular Accumulation of Long-Chain MTX-PG in Acute Lymphoblastic Leukemia Cells

**Figure 2:**
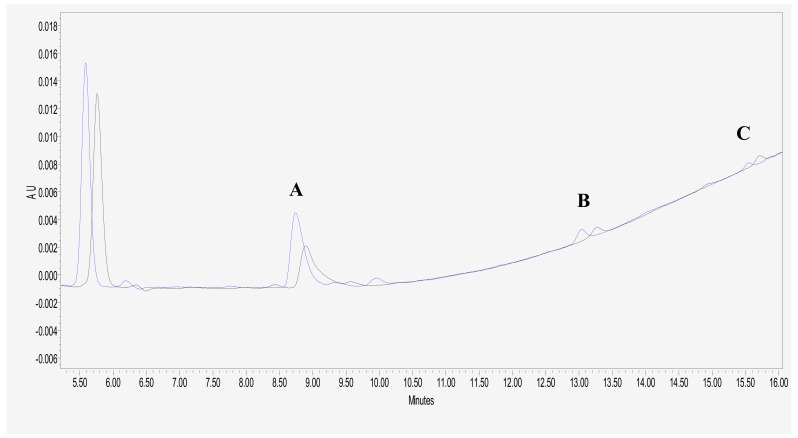
HPLC chromatograms of MTX-PG. Chromatogram illustrating MTX polyglutamate metabolites in CCRF-SB cell extracts after exposure of the cells to MTX alone (black line) and methotrexate plus 20 mM glutamic acid for 24 h (blue line). MTX-PG_5_ (**A**), MTX-PG_4_ (**B**), and MTX-PG_3_ (**C**).

**Figure 3:**
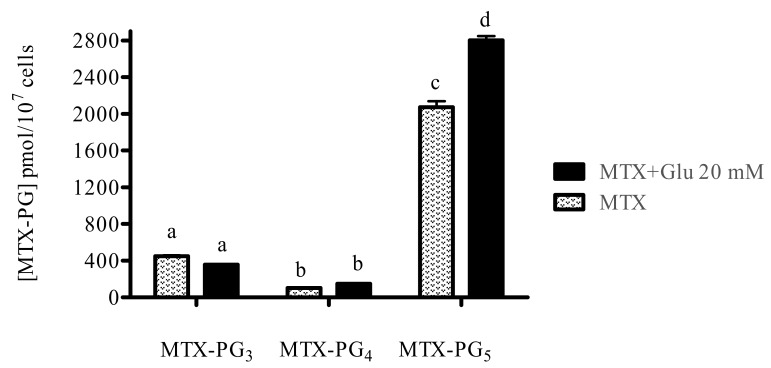
Quantification of MTX-PG_3-5_ levels by HPLC. Methotrexate polyglutamate levels in CCRF-SB cells after exposure to MTX (0.26 μM) or MTX (0.26 μM) plus 20 mM glutamic acid for 24 h. Data are expressed as the mean ± standard deviation. Different letters represent significant differences among treatments (*p* < 0.05).

To evaluate the pharmacological effect resulting from the combination of glutamic acid and MTX, we determined the intracellular accumulation of MTX-PG in the ALL cell line after simultaneous treatment with both drugs. Logarithmically growing CCRF-SB cells were treated with MTX (0.26 μM) and glutamic acid (10 or 20 mM) for 24 h (data not shown for MTX plus glutamic acid 10 mM treatment). Intracellular drug metabolites were determined by high-performance liquid chromatography as described in Materials and Methods. As shown in Figure 2 and Figure 3, the levels of methotrexate pentaglutamate (MTX-PG_5_) were significantly higher in cells treated with 20 mM glutamic acid compared with those of cells that were exposed only to MTX and MTX plus 10 mM glutamic acid.

### 3.3. Treatment with MTX or MTX/Glu Induces the Expression of the FPGS Enzyme and the GLAST Transporter

To test the effect of the different treatments (MTX or MTX/10 or 20 mM Glu) on the expression of FPGS and GLAST proteins, we investigated the presence of FPGS and GLAST proteins in treated and untreated CCRF-SB cells by immunocytochemical analysis.

**Figure 4:**
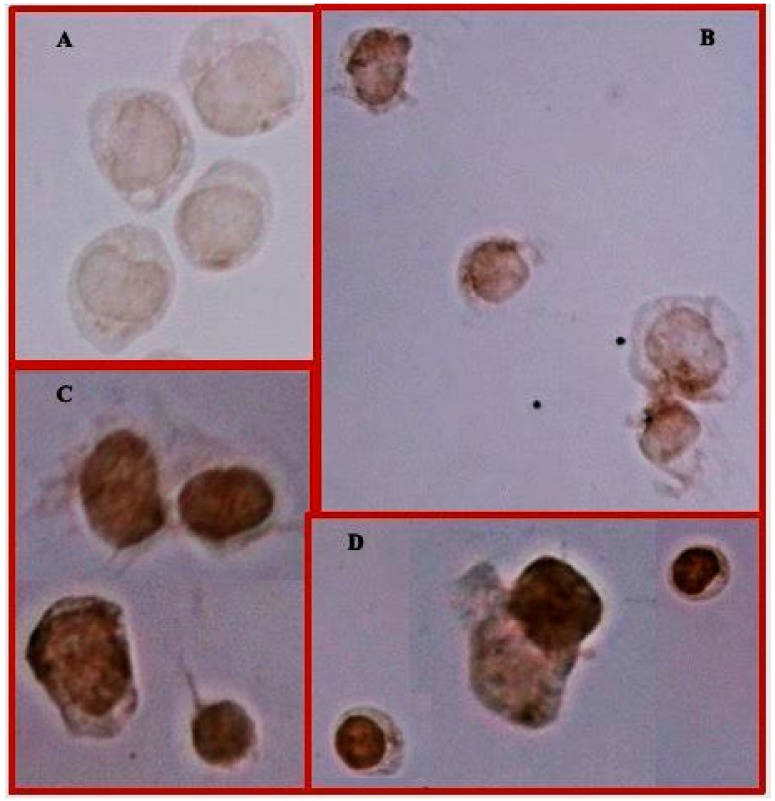
FPGS expression and cell distribution in CCRF-SB cells. Representative cells of FPGS protein immunocytochemical staining. Untreated cells (**A**), cells treated with MTX (**B**), MTX/10 mM Glu (**C**), or MTX/20 mM Glu (**D**). 40× magnifications.

**Figure 5:**
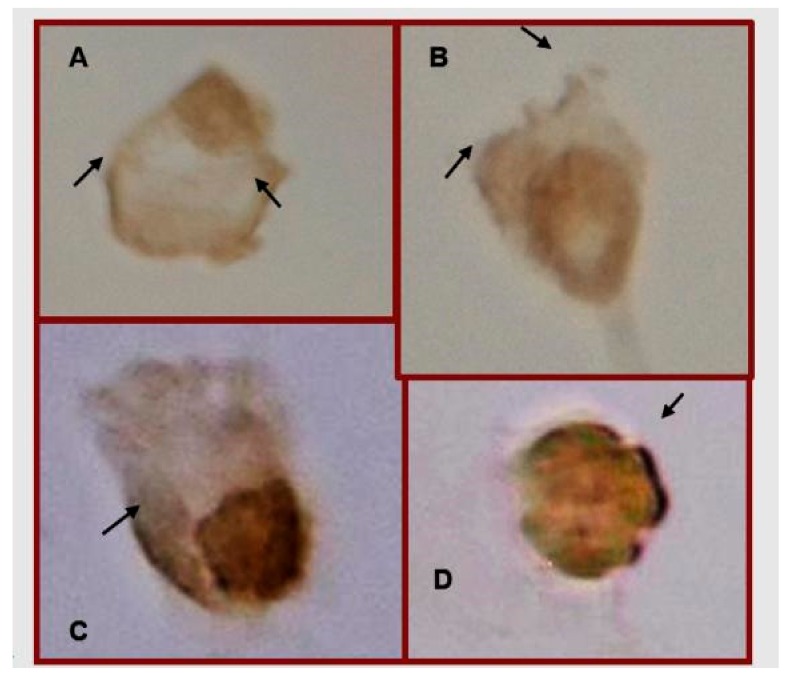
GLAST expression in CCRF-SB cells. Representative cells of GLAST protein immunocytochemical staining. Untreated cells (**A**), cells treated with MTX (**B**), MTX/10 mM Glu (**C**), or MTX/20 mM Glu (**D**). 40× magnifications. Arrows indicate the localization of GLAST in cellular membrane.

In all treated cells examined, the treatments induced an increase in the expression of FPGS and GLAST proteins compared to that of the control group (Figure 4 and Figure 5). Additionally, the immunocytochemical analysis indicated different FPGS staining zones. For example, predominant staining was observed in the cytoplasm of cells treated with MTX or MTX/10 mM Glu, and cells treated with MTX/20 mM Glu. We detected an increase in staining in the cytoplasm compared with that of the control cells or cells in the other treatment groups (Figure 4D).

### 3.4. The Combination Treatment of Glutamic Acid–MTX Increases Apoptosis in CCRF-SB Cells

**Figure 6:**
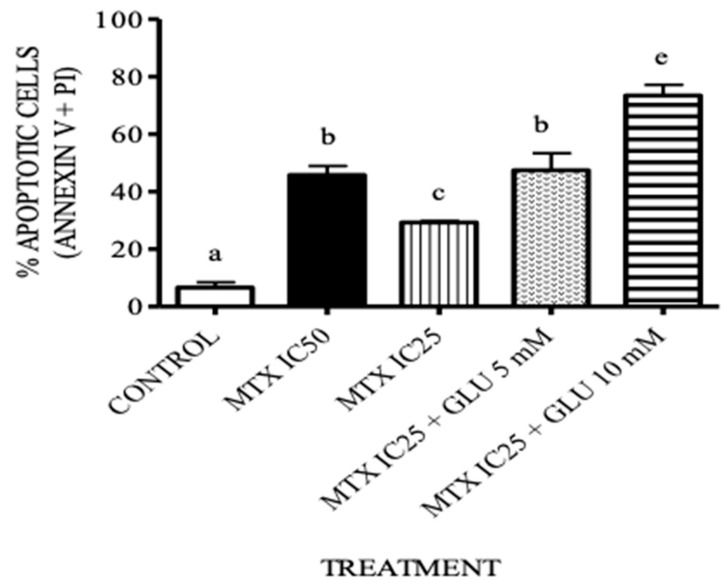
Glutamic acid increases the apoptotic effect of MTX in B-ALL cells. The percentages of cells undergoing apoptosis (annexin V^+^ + PI ^+^) were measured by flow cytometry after treating the cells with MTX or MTX+Glu for 24 h (see Materials and Methods for details). Different letters represent significant differences between treatments (*p* < 0.05). Glu: glutamic acid; MTX: methotrexate; IC_25_ = 0.13 μM; IC_50_ = 0.26 μM.

As shown in Figure 6, treatment with 0.13 μM MTX + 5 or 10 mM Glu significantly increased apoptosis in CCRF-SB cells (*p* < 0.05) compared with exposure to 0.13 μM MTX alone. The effect of the combination therapy with 0.13 M MTX + 5 mM Glu was equal to the response shown by treatment with 0.26 μM MTX, while MTX + 10 mM Glu increased the number of apoptotic cells significantly more than any other group.

### 3.5. Effect of Glutamic Acid–MTX Treatment on Glutamate Uptake

**Figure 7:**
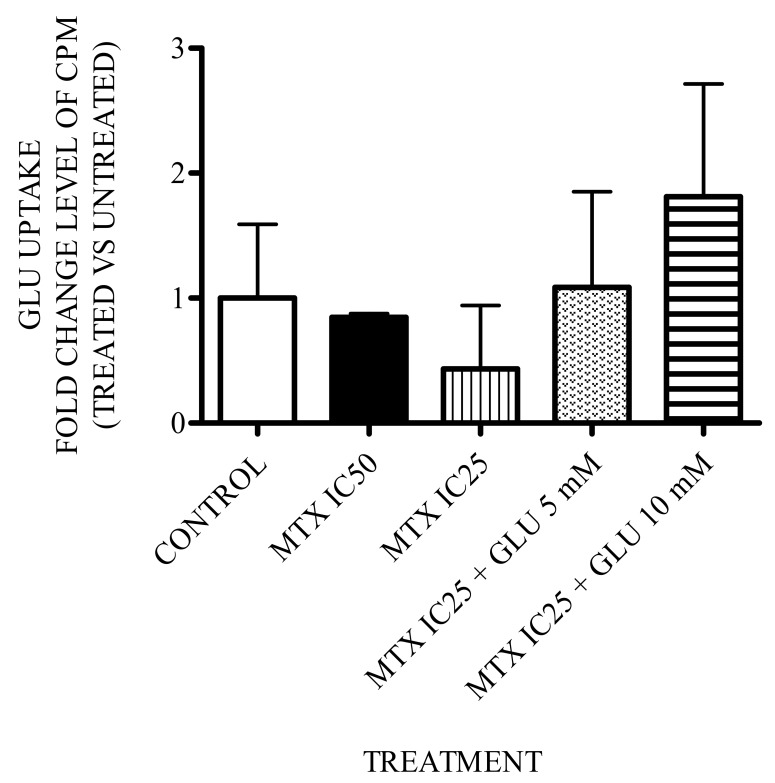
Glutamate uptake. Glutamate uptake is expressed as the fold change in cpm (counts per minute) detected in treated versus untreated cells. Glu: glutamic acid; MTX: methotrexate; IC_25_ = 0.13 μM; IC_50_ = 0.26 μM.

To determine whether the treatments (MTX IC_25_ or IC_50_ as well as MTX/5 or 10 mM Glu) would influence glutamate transport, we evaluated glutamate uptake by a radioactive assay. As shown in Figure 7, none of the treatments significantly altered glutamate uptake. However, there was a tendency to increase the transport of this amino acid inside the cell.

## 4. Discussion

Acute lymphoblastic leukemia is the most common type of cancer in the childhood population. Even though progress has been made in the treatment of ALL, the outcomes for a great number of children continue to be dismal. Therefore, novel treatment schemas are needed to resolve this issue. In this context, data from the present study indicate that co-treatment with glutamic acid (Glu) at concentrations of 10 and 20 mM plus the highly effective antileukemic drug MTX improves the cytotoxicity of MTX and promotes an increase in the synthesis of MTX-PGs in CCRF-SB (B-ALL) cells. The increase of MTX-PGs is the key to the antileukemic effect of MTX, which provides a better prognosis for patients with ALL, especially for patients whose lymphoblasts do not accumulate the drug as well [8].

This is the first time that MTX IC_50_ and IC_25_ values have been determined for the CCRF-SB cell line. This is also the first time the effect of the MTX plus Glu combination has been studied in vitro. Glu is not an anticancer agent, but when used at doses of 5 and 10 mM with 0.13 μM MTX, it enhances the cytotoxic effect compared to treatment with 0.26 μM MTX alone, which means that doses of MTX could be reduced. To investigate the reason for the enhanced cytotoxicity, we quantified MTX-PGs by HPLC as described by Barnes et al. in 1998, and we found that the cytotoxicity of the combination therapy correlated well with the increased levels of MTX-PGs, especially with MTX-PG_5_, because levels of MTX-PG_3_ and MTX-PG_4_ were lower. This is understandable since there is evidence that confirms that B-lineage ALL cells predominantly accumulate MTX-PG with 2–7 glutamyl residues [17]. In addition, there are reports that support that a concentration of 4 mM glutamic acid is enough to initiate the synthesis of MTX-PG [18]. However, in this study, we tested 20 mM Glu, which better enhanced the cytotoxicity of MTX (data not shown), which was reflected by a decrease in cellular viability. The action of Glu could be explained by its primary role as a source of energy for rapidly dividing cells such as cancer cells [19]. Additionally, previous reports have indicated that Glu increases the efficacy of anticancer drugs and decreases their toxicity towards normal cells [13], which is ideal for chemotherapy.

In our cytotoxicity analysis, the excitation spectra of the peak that appears near 5.6 and 5.8 min (Figure 2) is overlaid on the spectra of the MTX-PG calibrators; with this information and given the order of elution of this compound [20], we can suppose that it corresponds to methotrexate hexaglutamate (MTX-PG_6_). Furthermore, if the identity of the peak is confirmed in the future as the MTX-PG with six glutamyl residues due to its higher absorbance in comparison to the other MTX-PGs, this would be at odds with the above-reported findings in regard to the predominant MTX-PG in B-ALL cells. For these reasons, the combination therapy of MTX plus Glu could be indicated primarily for patients with B-ALL because B lymphoblasts synthesize predominantly MTX-PG_3-6_, and it can be used as a predictor of favorable treatment outcomes in childhood ALL [6].

Moreover, we investigated the expression of FPGS because it is the determinant enzyme for MTX-PG synthesis and accumulation, mostly in B-ALL compared with T-ALL and myeloid leukemia, in which its activity is twofold lower [21]. We observed a rise in the expression of this protein in cells exposed to the different treatments. The upregulated expression of FPGS could be linked to the increased level of MTX-PG_5_, which possesses the ability to block the enzymes 5-aminoimidazole-4-carboxamide ribotide (AICAR) transformylase and adenosine deaminase [22]. This leads to an increase in the concentration of AICAR monophosphate (ZMP) and adenosine monophosphate (AMP); both ZMP and AMP are able to activate the AMP-activated protein kinase (AMPK) pathway [23]. AMPK can phosphorylate the cAMP response element binding protein (CREB) [24]. It is known that CREB, along with the Sp1 protein and NFY-B, forms a multi-protein complex that is associated with the promoter region of the gene encoding the enzyme FPGS. In this way, CREB can regulate the expression of the mRNA and protein levels of FPGS as well as GLAST, which increases its expression in the same way as FPGS [8]. The increase in FPGS and GLAST through treatment with MTX + 10 mM Glu and MTX + 20 mM Glu is correlated well with the increase in MTX-PG_5_.

In addition, we evaluated whether the increase in Glu dose alters its uptake into the cell, but we found that there were no significant differences between any of the groups. However, in cells treated with MTX + 5 mM Glu and MTX + 10 mM Glu, there was a tendency to increase the uptake of Glu, which is related to MTX-PG synthesis and GLAST expression. This could explain the increase in Glu uptake. Therefore, all our findings seem to be related; Glu enhances MTX-PG synthesis, and MTX-PGs improve GLAST and FPGS expression. The increase in FPGS leads to MTX-PG synthesis, which is the key determinant of MTX action. Therefore, because MTX plus 10 mM Glu did not significantly enhance MTP-PG synthesis compared to MTX alone (data not shown), we decided to evaluate whether 10 mM Glu plus MTX even had an apoptotic effect to explain the decrease in cell viability shown in Figure 6. We used a flow cytometry Annexin-V assay to evaluate apoptosis and observed that 0.13 μM MTX + 10 mM Glu increased the number of apoptotic cells more than 0.26 μM MTX alone did. This suggests that Glu could act by another pathway leading to an anticancer effect [25], and due to this effect, MTX doses might be reduced.

## 5. Conclusions

In conclusion, we have provided evidence that Glu in both 10 and 20 mM concentrations sensitizes CCRF-SB (B-ALL) cells to the cytotoxic effect of methotrexate. The sensitization of CCRF-SB cells to methotrexate by Glu could be through increasing the synthesis of MTX-PG_5_, which leads to the increase in the expression of the enzyme FPGS and GLAST, leading to polyglutamation and Glu uptake, respectively. These events result in decreased cell viability. In perspective, and with the aim to validate these results, it is necessary to test the combination therapy in vitro with samples of patients with B-ALL and other B-ALL cell lines.

## Figures and Tables

**Table 1:** Drug combinations to evaluate MTX cytotoxicity and Glu uptake.

	Control	Group 1	Group 2	Group 3	Group 4
MTX IC_25_	----	+	----	+	+
MTX IC_50_	----	----	+	----	----
Glu 5 mM	----	----	----	+	----
Glu 10 mM	----	----	----	----	+
